# Safe management of ophthalmic health care waste

**Published:** 2021-07-20

**Authors:** Astrid Leck

**Affiliations:** 1 Assistant Professor and Microbiologist: London School of Hygiene & Tropical Medicine, London, UK.

**Health care waste must be collected, handled, stored and disposed of in such a way that it poses no risk to patients, personnel, or the public.**

Hazardous waste relevant to eye health includes:

- **Sharps waste.** Used or unused sharps (e.g., hypodermic, intravenous or other needles, scalpels, broken glass)
- **Infectious waste.** Waste that poses a risk of disease transmission, e.g., waste contaminated with blood and other bodily fluids, laboratory cultures, and other materials that have been in contact with infected patients.
- **Pathological waste.** Human tissues, organs, or fluids.
- **Pharmaceutical waste.** Medicines and items contaminated by medicines.
- **Chemical waste.** Disinfectants, solvents, batteries, broken thermometers or blood pressure gauges, and laboratory reagents.

**What is health care waste?**

Health care waste is the term used to describe waste generated by health care practices (both within clinical settings and by health practitioners in the community).

Incorrect management of health care waste can result in harm to patients, staff members, and the community. It can result in the unintentional release of pathogens (including drug-resistant organisms), pharmaceutical drugs, and toxic pollutants) into the environment. Disposal of untreated waste in landfill sites may contaminate water sources if the landfill is poorly constructed. The release of effluent water containing chemical disinfectants or commonly used laboratory chemicals (such as staining reagents used in diagnostic microscopy) can also pollute drinking, surface, and ground water sources.

Incineration can be costly and polluting, and improper incineration practices can result in low temperature burning. Low temperature burning will not destroy sharps waste and may expose health care personnel and the community to toxic compounds released from burning plastic and expired medicines.

Additional hazards can occur if people are scavenging at waste disposal sites and during the handling and manual sorting of hazardous waste from health care facilities. Waste handlers are at immediate risk of needle-stick injuries and exposure to toxic or infectious materials. Waste collection and storage areas on health care premises must be secure and access by unauthorised persons prohibited.

Lack of awareness about the health hazards related to health care waste, inadequate training in proper waste management, an absence of waste management and disposal systems, insufficient financial and human resources and the low priority given to the topic are the most common problems. Many countries either do not have appropriate regulations, or do not enforce them.

## How to manage waste

Good practice begins with minimising the volume of waste generated in the health care setting through careful selection of consumables and processes, in combination with safe handling and the segregation of waste where it is being generated. Equally important is raising awareness and working towards improving local practice to attain national and internationally recognised safety standards.

**Figure 1:**
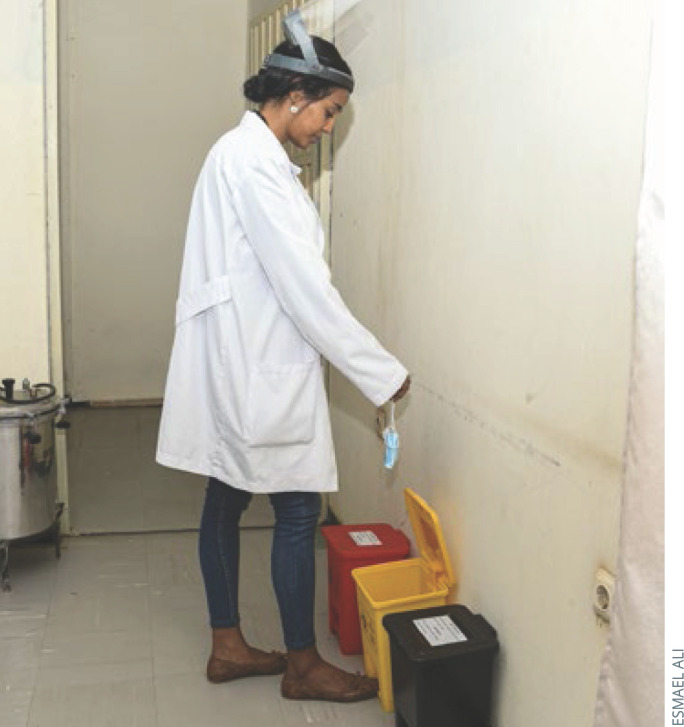
Bins for waste collection must be clearly labelled and ideally hands-free, pedal operated and colour coded. **ETHIOPIA**

Factors such as transportation, location and budget could influence how a health care facility will manage its waste. However, even in health care settings which have limited resources, it is essential that managing health care waste is viewed as a priority.

As a first step, it is important to determine if there are any national policies in place and, then based on this information, to create guidelines which are appropriate to both local facility and community needs and health care waste categories.

In larger hospitals, it is helpful to set up a waste disposal committee; in a smaller clinical setting a member of the infection control team will take on this responsibility in partnership with estates or clinic management. A survey or audit must be carried out to assess the both the volume and categories of waste generated by the eye care facility service before a waste management plan can be created and implemented by hospital/clinic management.

Health care waste must be collected, handled, stored, and disposed of in such a way that it poses no risk to patients, staff members or the public. Waste management protocols are needed for the collection and safe storage of health care waste both at ward level and storage in other areas within the eye hospital, eye clinic or health care premises. At a minimum, hazardous waste must be contained and separated from general waste. The use of colour-coding of waste packaging to clearly identify and categorise the type of health care waste before disposal simplifies this process. Table 1 gives the colour, type of container and collection frequency recommended by the World Health Organization (WHO) for different types of waste (Table 1).

**Table 1:** Colour coding, container type and collection frequency of waste recommended by the World Health Organization (WHO)

Waste categories	Colour of container and markings	Type of container	Collection frequency
**Infectious waste**	Yellow with biohazard symbol (highly infectious waste should be additionally marked **HIGHLY INFECTIOUS**	Leak-proof strong plastic bag placed in a container (bags for highly infectious waste should be capable of being autoclaved)	When three-quarters filled or at least once a day
**Sharps waste**	Yellow, marked **SHARPS** with biohazard symbol	Puncture-proof container	When filled to the line or three-quarters filled
**Pathological waste**	Yellow with biohazard symbol	Leak-proof strong plastic bag placed in a container	When three-quarters filled or at least once a day
**Chemical and pharmaceutical waste**	Brown, labelled with appropriate hazard symbol	Plastic bag or rigid container	On demand
**Radioactive waste**	Labelled with radiation symbol	Lead box	On demand
**General health-care waste**	Black	Plastic bag inside a container or container which is disinfected after use	When three-quarters filled or at least once a day

All staff members who come into contact with health care waste must be trained to know how to classify, handle and dispose of it safely and how to deal with any spillages. Anyone handling waste should have access to appropriate personal protective equipment (such as thick rubber gloves and aprons) that will help them to carry out their duties safely.

The WHO’s standards for health care waste disposal include the use of incineration and protected pits.^1^^,^^2^ On-site incineration is recommended for microbiological, pathological and anatomical waste. Laboratory waste known to contain pathogens should be autoclaved to render it safe before it is removed from the laboratory.

**Figure 2:**
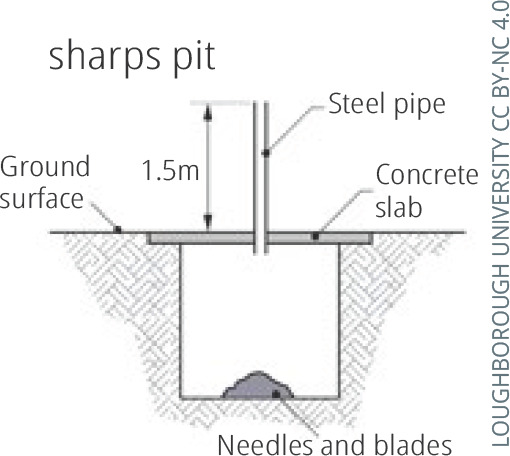
A protected sharps pit

Alternative methods of waste disposal may be important in low-income settings that are unable to support high-temperature incineration practices. The use of **protected pits** is one example. These are deep, square pits within hospital premises that are topped by a reinforced concrete slab which is wider than the pit. The concrete slab has a smaller hole covered by a smaller piece of concrete which can be removed when filling the pit. Another option, recommended for sharps, is to insert a steel pipe (Figure 2) in the main concrete lid. Once the pit is full, the pipe (or the access hole) is permanently sealed.

**Figure 3:**
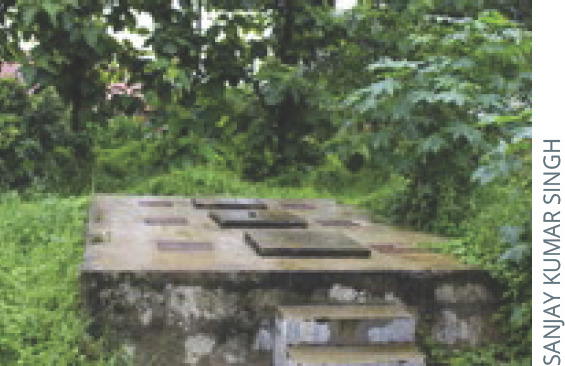
Protected pits with concrete covers, Nepal

**Waste chemicals** need to be safely stored until they can be collected by specialist disposal companies. If such a service is not available, there may be alternative options, for example, filtering reagents through charcoal to inactivate chemicals, after which the liquid can be poured away into a drain. Alternatively, liquid reagents can be poured into a leakproof container of highly absorbent clay or crystal pellets (for example, those used for domestic cat litter). Used charcoal filters and pellets are hazardous waste. If hazardous waste is to be buried, it must be contained within a leak-proof container prior to storage in a protected pit.

**Microbiological waste and sharps** pose the greatest infection risk. Sharps are any medical device contaminated with blood, bodily fluids, or tissue and which can cause lacerations or puncture wounds. Safe handling involves correctly using and then discarding sharp items – at point of use – in specialised, robust containers which can be sealed for collection and disposal. Sharps containers must only be filled up to the indicated line (three-quarters full) and never overfilled; protruding sharps will compromise safe closure of the container. Heavy duty gloves must be worn by anyone who handles or transports these containers.

**Figure 4:**
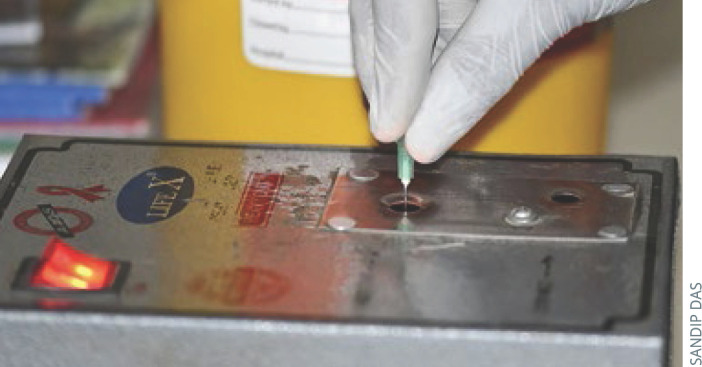
An electrically powered needle destroyer, Nepal

If incineration is not available, a full sharps plastic container can be filled with plaster of Paris, or something similar, to encase the sharps waste within the container, creating a solid mass that can be disposed of safely or buried. Automated machines which destroy needles by burning or cutting them can be used to render needles unusable and prevent needlestick injury (Figure 4).

**Make your own sharps container**

**Figure 5:**
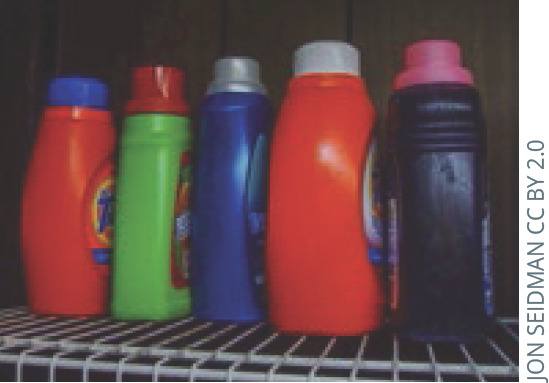
Sharps containers can be created using everyday items such as sturdy plastic detergent bottles

Commercially produced sharps containers are expensive. You can make a sharps container out of an old infusion bottle or a tough plastic laundry detergent bottle (Figure 5). It needs to have a screw top lid and must be clearly labelled as containing hazardous material. Once the container is three quarters full, it needs to be sealed with heavy-duty tape and either disposed of by incineration or buried in a protected pit.
